# Play in Elephants: Wellbeing, Welfare or Distraction?

**DOI:** 10.3390/ani10020305

**Published:** 2020-02-14

**Authors:** C. Elizabeth Webber, Phyllis C. Lee

**Affiliations:** Behaviour and Evolution Research Group, Psychology, Faculty of Natural Sciences, University of Stirling, Stirling FK9 4LA, UK; redwellies271@hotmail.com

**Keywords:** elephant play, captive wellbeing, comparative play rates, early development

## Abstract

**Simple Summary:**

Animal play is a subject of great interest and some enduring controversy. Why do animals play, when do they play and if they do not play much, does this indicate that they may be physically or emotionally stressed? We explore these questions for elephant calves ranging in age from birth to five years old, and we compare play in captivity with that observed in the wild for two species. Against our general expectation that calves might play less play in captivity, we found that wild elephants spent the least time in play, probably because wild calves have to solve other social problems and be on the move constantly in order to find enough food, escape from predators, and keep up with their mothers and other relatives. Play is a diverse and subtle potential indicator of wellbeing for young animals, and we suggest that its presence needs to be interpreted with caution as it could represent either a distraction from a constant or unchallenging environment or provide arousal. Play appears to act as a behavioural mechanism for creating physical and social challenges for elephants of all ages, irrespective of their environment.

**Abstract:**

We explore elephant play behaviour since (a) play has been proposed to represent a potential welfare indicator; and (b) play has been associated with long-term survival in the wild. We categorised play into four types, and investigate both social (gentle, escalated-contact) and non-social (lone-locomotor, exploratory-object) play from observations made on wild (Asian N = 101; African N = 130) and captive (Asian N = 8; African N = 7) elephant calves ranging in age from birth to five years. Social play was the most frequent type of play among immature elephants, accounting for an average of 3%–9% of active time. Non-social play accounted for an additional 1%–11% of time. The most time spent in play was seen in captive Asian calves, particularly at the ages of 1–6 months, while wild African calves spent the least time in play overall, even though they had the greatest number and most diverse range of play partners available. We assessed calf energetics using time spent suckling, resting, moving and independent feeding. Time spent playing was unrelated to time spent suckling but negatively associated with time spent independently feeding. There were no associations with time spent moving or resting. Maternal energy via lactation was unrelated to play early in life, but energy acquired independently may constrain or enable play. Play, while a potential indicator of compromised welfare for many species when absent, can act as a highly stimulating activity for captive elephants in the absence of other forms of arousal.

## 1. Introduction

Play is a complex behaviour with a variety of benefits as well as costs for playing organisms. Play is widely distributed across taxa, from invertebrates to fish, birds and mammals [1]. The quest for a single ‘function’ of play has been fruitless. Rather, recent approaches have concentrated on two key areas: (a) what does play accomplish for a playful organism in its current context [2], and (b) what does play effect over the life course of the organism. These distinctions, between juvenile adaptations and ‘scaffolding’ [3], might be especially important when examining play behaviour in captive contexts, as these differ markedly from the animal’s wild social and ecological environment.

Similarly, formally defining play remains problematic [1]. The working definition of play uses five simultaneous criteria: (a) lack of functionality; (b) pleasurable and voluntary; (c) exaggerated; (d) repeated; and (e) occurs in the absence of stress [1,4]. We would add, as suggested by Byosiere et al. [5], (f) play in a social domain requires communication and exchange, or meta-communication [6,7].

Variations in play rates among birds and mammals are a function of age, sex, season, individual personality, and social and physical environment, making comparisons even within a species and social or environmental context difficult to interpret. However, elephants are a taxa where play may be especially revealing of mental state or affect since play is diverse and frequent, and occurs throughout their lives being seen even amongst the oldest ages [8]. During immaturity, elephants show sex-specific play partner choices with males seeking out novel, non-familiar play partners while females direct play towards vulnerable family infants [9,10]. Thus, the number, diversity and availability of play partners might affect how often elephants will play [8,9,10]. Additionally, play early in development has been related to long-term survival in elephants, with high play rates indicating some aspect of individual quality while reduced play rates were associated with reduced growth rates and, consequently, increased mortality [8,11]. In brown bears [12,13], more playful cubs had higher juvenile survival, controlling for maternal condition effects, suggesting a direct association between play, cognitive flexibility and survival skills. These association between play and “the unexpected”, whether social or physical, may endow organisms with behavioural resilience in the face of unpredictable changes [13,14,15,16,17]. Play is also important for self-assessment of physical and social abilities [18] including relative size and rank [19]. Juvenile rats [20] and hamsters [21] that exhibited play were more socially competent as adults [22]; for example, among captive mink, more play was associated with greater male mating success [23].

Play has demonstrable costs. The first of these is energetic, with play reducing growth rates in pronghorn fawns [24] and Assamese macaques [25]. The energy costs of play, even if relatively small for a play event [26], can accumulate if considerable time is spent in play. In macaques, slower growth was traded for greater facility in solving foraging problems among more playful immatures [25]. Social exchanges as part of play can also represent risks; for example, disease transmission increased due to play contact in chimpanzees [27], while being distracted from vigilance can lead to death from predation [28] or injury [29]. 

Play normally occurs only once more pressing physiological needs have been satisfied and tends to diminish in the repertoire when individuals are under energy limitations [30,31], physical stress from inclement weather [32] or stressors such as weaning. During weaning, individuals experience both a loss of energy and of attention from the mother (e.g., domestic pigs [33]; cattle [34]), resulting in reduced play. Supplementing energy via artificial feeding can restore or enhance rates of play, even in wild species (deer [35]; meerkats [36]). As independent feeding comes to replace milk energy, this acquired energy then has to sustain both growth and play [24,37]. It can thus be predicted that play will have to be balanced against other activities also requiring energy, such as foraging and travel, and will be reduced in frequency when stressors such as weaning occur.

### Play Behaviour: Development and Welfare?

While much debate revolves around “why” play (its evolutionary and functional components), here we are concerned with how play in captive elephants develops and is expressed, and how it compares with that seen in wild calves. Play behaviour is linked to positive emotions and frequently accompanied by pleasure [38,39,40,41,42]. One challenge for ensuring good welfare lies in providing just such positive experiences—including pleasure, e.g., excited playfulness or affectionate sociability—rather than merely ensuring the absence of negative experiences [43]. Mellor’s [44] concept of a “life worth living” for human-constrained animals opens the opportunity to use play as an indicator of high-quality life experiences, which are in short supply for most captive elephants (ill health, foot problems, obesity, dystocia, stereotypies, truncated lifespan: [45]). Captive contexts, while meeting many short-term needs and thus promoting some forms of play [41], may still be unable to deliver the range of social opportunities and physical environments that are an essential part of play experiences [15,16,17]. Furthermore, if play typically occurs when individuals are free from stress and in the absence of threats, and when basic short-term needs have been met, then play will be reduced or dropped entirely when conditions become challenging or constrained. Play, from keeper reports, appears to be rare in captive elephants at 2%–4% of active time [46]. That so few facilities (less than 20% in the US [47]) have juvenile elephants available as interactive partners emphasises the potential significance of understanding play in relation to other processes such as births and deaths affecting captive welfare [48].

Despite play’s potential significance in elephant development [8,9], its utility and validity as an indicator of welfare is under debate [49,50] and we need to be cautious when interpreting play types and frequencies as a positive welfare indicator [51]. Many species play more in captivity than in the wild, due to abundant energy availability and intake and few competing activities [1], while some play less due to limited opportunities or partners [41,52]. In addition, play has been associated with conditions of severe stress (e.g., riding school horses, [53]). Play might thus act both as a coping mechanism for individuals with compromised welfare as well as a signal of exploration, exuberance, and pleasure [1].

Our first objective was to investigate whether captive and wild elephant calves engaged in the same types of play and whether these varied as a function of age, sex and species. These basic descriptions are rare for captive elephants (but see [54]), and comparisons between wild and captive calves will shed light on the range of play behaviours possible, when these are observed during development, and whether they have any utility as indicators of wellbeing. Understanding the levels of variation in activities and behaviours between wild Asian and African elephants will also enable a greater understanding of the variance that we see in captivity. 

We hypothesised that wild calves would play more than captive calves, at least in part due to the greater availability and diversity of partners in wild fission-fusion elephant societies [8]. If partners were less available or less diverse, we hypothesised that lone play would replace social play; thus, captive calves were predicted to use active behaviours such as lone play or object play to compensate for relatively few social partners and social interactions in captivity (see [29,41]). It is, however, important to distinguish between a lack of novel stimulus as opposed to the simple lack of play partners. All the captive calves lived in reproductive units with a variety of peers of the same and different ages, so each had potential play partners available; unlike wild calves, however, these peers seldom changed and thus were constant and well-known, possibly unchallenging, partners. Predictions tested here were: (a) age and sex patterns in play will be similar for both species in the wild, (b) captive calves will exhibit more lone play than social play, and (c) social play can be used as an indicator of positive affect and wellbeing. Partner choice could not be tested directly, as it was either constant in captivity or difficult to assign partner identity for wild Asian calves due to limited visibility. It was however possible to briefly explore whether group size and composition influenced elephant calf play behaviours. Finally, we examined the relationships between play, suckling interactions in relation to weaning, and energetic activities such as moving and independent feeding as a function of calf age, sex, species and context (wild/captive).

## 2. Methods

### 2.1. Classification of Play Types

Since the costs and potential benefits of the main play types (social and non-social) may vary, we examined these separately and used definitions of elephant play from Lee and Moss [8,10] and Poole and Granli [55] (see Table 1). No study of wild Asian elephant calves has yet described play in detail, so the terms and contexts used here will need further exploration for other play types and interactions in additional populations of both species. Our basic distinction is between social play, which involves interactions with others, and non-social play, where play is neither with, nor directed towards, conspecifics [10,56]. Social play included both escalated (high energy, “rough and tumble”) and gentle-contact play with conspecifics. Sparring is the most common form of social play in elephants, particularly between males [57]. 

Elephants engage in many forms of lone and object play, presumably for pleasure [55]. Individuals gather information about their environment through exploration and use object play to discover what can be ‘done’ with the object (including repeating actions which have similar outcomes). A distinction between object play and exploration was difficult to make in practice, as these behaviours lie along a continuum for species like elephants. Environmental exploration includes throwing/kicking of dust, mud, water, and vegetation, or approaching, chasing and vocalising at objects (birds, monkeys, other species) in the environment, while environmental play typically occurs with exaggerated movements of the head, trunk and body and often with trumpeting vocalisations [55]. We considered exploration as play when it was repeated, exaggerated and accompanied by postures or vocalisations seen in play. 

Likewise, active solicitation behaviours (alone or in combinations) are often observed, leading to social play, and we suggest that these are signals of intentions to play [5,6]. Signals may include tusking the ground, kneeling invitations, waggling the head, raising trunks (spar invitation) and curling the trunk over tusks with the head back [55]. Head high, and movement with floppy-head while walking and erect body postures indicating playfulness, can signal over long distances and any resulting interactions between signaller and recipient are almost never aggressive. In order for older and larger elephants to elicit play in younger, smaller individuals, one animal may self-handicap [16], for example by lying down or getting down on their knees. This not only allows the younger animal to have more physical contact and to even climb on partners but also makes them appear less intimidating [55]. 

### 2.2. Observational and Analytical Methods

Elephants can be recognised individually by natural markings on ears such as size, shape, vein patterns, notches, holes and folds [58]; from bodies, such as back shape, scars or other marking; distinctive tails including varying lengths, kinks, hairlessness or even unusual hair colour; and tusk and tush (small tusks) size and shapes if present. 

Data on Asian elephant calves were collected by CEW (C.E. Webber) between 2011 and 2013 in Uda Walawe National Park, in southern central Sri Lanka (latitudes 6°25’–6°34’ N and longitudes 80°46’–81°00’ E) in collaboration with the Uda Walawe Elephant Research Project (UWERP). The park is an area of approximately 308 km^2^ and is a highly seasonal environment, covering dense riparian forest, tree plantations, secondary forest, dense scrub, and both tall and open grassland areas. The elephant study population in Uda Walawe was estimated to be between 804 and 1160 individuals in 2011, with individual identification records for 286 adult females and 251 adult males, and a density ranging from 102 to 116 adult females per 100 km^2^. A total of 101 calves (39 male, 56 female and 14 of unknown sex) with reliably estimated birth dates and known mothers were studied up to approximately five years of age during the dry seasons of three years. Calves were sampled using 10 min video recordings which were subsequently transcribed as 5 min interval-sampled observations (“scans”) of calf and mother activities (Table 2). These scans were only included when scored as “good observations”; e.g., the calf was clearly visible (had not walked behind vegetation, the mother or others). Thus there would typically be 3 scans from each 10 min video sample for each individual calf (min = 2, max = 17 for one 2-hR video taken opportunistically at the start of the study in the presence of a large group). 

Data on African elephant calves were collected by PCL (P.C. Lee) and CJM (C.J. Moss) between 1979 and 1984 in Amboseli National Park, Kenya (02°38’29” S 37°14’53” E), a 390 km^2^ protected area on the border between Kenya and Tanzania. The elephants move over a savannah ecosystem of 3000–8000 km^2^ and have been observed as individuals since 1972 (Amboseli Elephant Research Project-AERP). Relevance and continuity of these detailed observations have been validated by long-term daily observations of activities (47 years; AERP long-term records). The study population at the time was around 700 elephants with 162 calves of less than six years old and 94 calves were born during the study period [9]. Focal animal and scan data from November 1982 to November 1984 are analysed here, and observations were evenly balanced across months and seasons. For the calves included in our analyses, month and year of birth are known and these calves have subsequently been tracked by members of AERP for the past ~40 years. Calves and mothers were sampled using checksheets designed for a 60 min continuous focal samples of neighbours and interactions. Activity scans were made at 5 min intervals throughout the hour to minimise dependence between successive intervals (Table 3). This interval was determined as optimal for detecting infrequent behaviours, while minimal for autocorrelation between successive samples, using comparisons with continuous records (see [59]).There were typically 13 instantaneous interval scans for each 60 min focal sample (min = 7, max = 17 for one 90 min focal recording sibling suckling). If focals were truncated due to loss of visibility or extended to allow for the completion of records on bouts of specific behaviour (suckling, aggression), only the number of valid interval observations were used for calculations of percentage of observations. 

The ethogram originally used for the wild African calf study was harmonised by CEW and PCL for the studies on wild Asian and captive calves [60]. Inter-observer reliability was assessed between PCL and CEW on a sample of 31 videos of wild Asian calves in 2016 to ensure that play was consistently recorded, with 89% concordance in codes, using Cohen’s Kappa Coefficient. 

Captive elephants were observed between 2010 and 2014, using the same focal sampling regime and ethogram at three facilities in the UK that had successful breeding programmes. All day observations on focal calves were made for six captive Asian and five captive African calves at monthly intervals from birth until 18 months of age. During daily focal calf observations, scan samples of activities were made at 5 min intervals (median N scans per day = 69, min = 3, max = 248). All calves, but one, were mother-reared and the groups had experienced no transfers or additions other than births, although two mothers were euthanised. Three calves died at birth and were not observed. One Asian calf born before the study started was also sampled at 13–18 and 19–24 months of age along with the data being collected simultaneously on younger calves (Table 4). Additional scan samples were made on individuals every 25 min to enable an assessment of activities and interactions of four previously unsampled juveniles (born before the study started) in the 3–5 year age category.

The percentage of good observations, where the calf and its activities were clearly visible, (hereafter called percentage time) spent in play, suckling interactions, or maintenance activities such as independent feeding, moving, and resting were calculated for each individual calf out of the total N of scans per day (from daily 5 min interval scans for captive calves, from 5 min intervals during videos on wild Asian calves, and from 5 min intervals during 60 min focal samples for wild African calves). Means and 95% confidence intervals are presented for non-log transformed values as medians for rare events will be zero. Percentage of observations for each calf on each observation day were assigned to a six-month age category up to 24 months, after which these were coded as 3–5 years. 

The effects of age, sex, species and context (wild/captive), and the interactions between these variables, on time spent in play (total, social, non-social play) were assessed using General Linear Mixed Models (GLMM in SPSS v21 ©IBM). GLMMs were run using variance components with Satterthwaite method (which reduces degrees of freedom) and robust estimates of covariance. Percentage of observation time spent in each play type was tested for normality. Due to high skew and many zeros, lone and social play could not be transformed when not normally distributed while major activities and percentage of observations of total play were normally distributed. All models showed significantly good fit (Supplementary Materials). No wild calf was observed more than 5 times with a median of one observation at each age, while captive calves were observed between 4 and 8 different times at each age. Individual calf ID was used as a random variable to control for repeated samples on the same calf. A sample of 10 or more is expected for fitting random variables; while this is not the case for captive calves within species, we were careful in our model design, limiting the number of interactions between or within factors and carefully assessing the contribution of individuals to the overall model fit. Full models were simplified by removing factors sequentially starting from the least influential (highest *p* value or *ß* parameter value closest to zero). If removing this term caused a decrease in the explanatory power of the model (using the overall model fit *F* value), the term was reinstated. Non-significant interaction between fixed factors were removed first, followed by non-significant main factors. Each dropped term was then added back into the final minimal model to check that significant terms had not been wrongly excluded. We present model fit (*F* value and significance) from the final model only. The parameter values (coefficient *β*) with 95% confidence intervals for significant effects are provided in Supplementary Materials (Table S1 for total play, Table S2 for social play and Table S3 for lone play). Pairwise comparisons for variables within significant main effects were carried out for sex (male, female), context (captive, wild, by species, *N* = 4) and age-categories (1–6 months, 7–12 months, 13–18 months, 19–24 months, 3–5 years, *N* = 5). Post-hoc comparisons were adjusted for multiple tests using least significant difference and significance (*p*) associated with pairwise comparisons represents that adjusted for the N of comparisons. When sample sizes were very small and skew was extreme, pairwise comparisons were limited to species and captive/wild only. While GLMM analysis is robust to violations of normality [61], results should still be treated with caution due to the very small N of calves in captive samples. Residuals were examined for normality in final models. Covariance in maintenance activities was examined using non-parametric Spearman correlation coefficients run on untransformed percentage of time for each calf’s observations in each category, with a Bonferroni correction for multiple comparisons. 

The data presented here are constrained by factors including visitor effects, management schedules, inconsistencies in calf visibility, seasonal variation in food availability for the wild Asian calves (see Lee and Moss [8] for a discussion of seasonality in the Amboseli data), and the small sample sizes in captivity at each age and by sex. Due to these limitations, analyses on captive calves should be considered as representing individual patterns. Individuals plots of play for individual captive calves sampled from birth to 18 months are presented in the Supplementary Materials (Supp Figures S1 and S2). We present overall statistical trends and plot values to illustrate variation between species and contexts and to enable subsequent further studies. 

### 2.3. Ethics and Research Permission

All observations were non-invasive in both the wild and captivity and were made with full local research and ethical permissions. Ethics for the study were approved by the University of Stirling’s Psychology Ethics Committee (CEW for her study) and the Animal Welfare and Ethics Research Board (PCL for the long-term study), University of Stirling. Permission for field research in Sri Lanka was granted by the Department of Wildlife Conservation. Research clearance was granted by Kenya National Parks (now Kenya Wildlife Service) and Office of the President, Republic of Kenya. Permission was granted to study calves at NEZS Chester Zoo, ZSL Whipsnade Zoo and Howletts Wild Animal Park. 

## 3. Results

### 3.1. Play Types by Context

**Figure 1:**
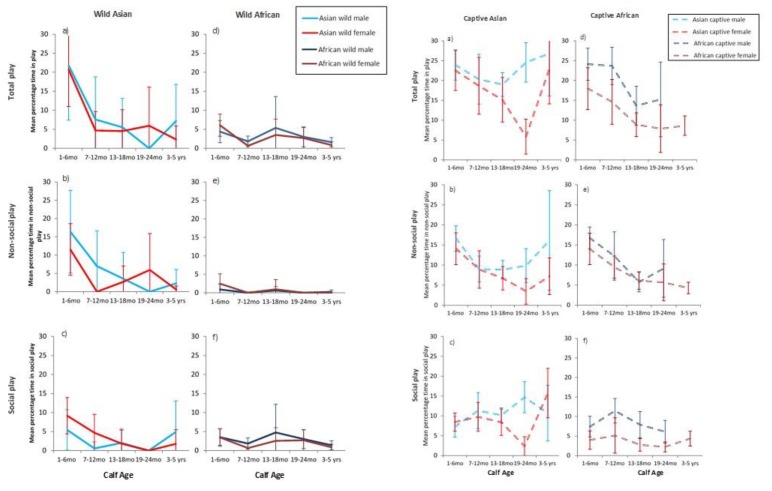
Mean percentage of observation time in play (±95%CI) for wild and captive calves across ages and by sex.

Calves engaged in both social and non-social play, with between 3% and 20% of time spent in play activities by individuals (Figure 1). Context had an overall significant effect on time spent in all forms of play, with captive calves of both species playing significantly more than wild calves (F_3,58_ = 26.03, *p* < 0.001). The mean percentage of time that calves engaged in play was highest at 1–6 months of age compared to all other ages (F_4,492_ = 11.61, *p* < 0.001, see Figure 1) and this peak and subsequent decline was particularly marked for wild Asian calves (*ß* = 12.31, *Adj p* < 0.001). Calf sex had no overall effect on play (*p* = 0.089). There was, however, significant individual variation in time spent in play (Var (ID) = 24.98, *p* = 0.003) (full GLMM model in Supplementary materials). 

In summary, the mean percentage of total time spent in play by either species in captivity was significantly greater than in the wild overall, and captive Asian calves engaged in significantly higher levels of play than did all other calves especially at the youngest ages. Wild Asian calves also spent significantly more time in play than did wild African calves. 

For social play, there were again overall significant effects of contexts (F_3,37_ = 20.28, *p* < 0.001). Captive Asian calves again engaged in significantly higher percentages of social play than did calves from all other contexts (*ß* = 11.30, *Adj p* < 0.001; Figure 1). Pairwise contrasts found significantly higher percentages of social play for captive African by comparison to wild African calves (*ß* = −3.27, *Adj p* = 0.01). Neither age nor sex affected rates of social play overall (see Supplementary materials), and although age and sex did not interact significantly (F_4,513_ = 1.02, *p* = 0.399), an interaction was found between calf sex and context (F_12,384_ = 2.19, *p* = 0.012); pairwise contrasts suggested that males at all ages played more than females, and that this effect was especially marked for captive contexts (see Figure 1 and full model in Supplementary materials). There was no significant contribution of an individual to these patterns in social play (Var (ID) = 2.86, *p* = 0.132).

As would be expected from overall means (see Table 5), the percentage of time spent in non-social play differed by context (F_3,66_ = 18.15, *p* < 0.001, Figure 1). Non-social play in wild African calves was significantly lower than in all other contexts (*ß* = 13.85, *Adj p* < 0.001) while wild Asian calves played non-socially significantly less than both captive Asian (*ß* = −6.36, *Adj p* < 0.001) and captive African calves (*ß* = −3.9, *Adj p* = 0.027). There was an overall effect of age on percentage of time in non-social play (F_4,528_ = 18.54, *p* < 0.001), and an interaction between age and context (F_12,555_ = 3.30, *p* < 0.001). Captive calves engaged in non-social play significantly more especially when aged 1–6 months by comparison to their play at other ages (*ß* = 8.63, *Adj p* < 0.001). No significant differences were found between the sexes overall (*p* = 0.122) in time spent in non-social play. However, as with total play, there was a marked contribution of individual to these patterns (Var (ID) = 12.35, *p* < 0.001). There were no direct correlations between social play and lone play in any contexts (see also Figure 1).

### 3.2. Energetics and Play

**Figure 2:**
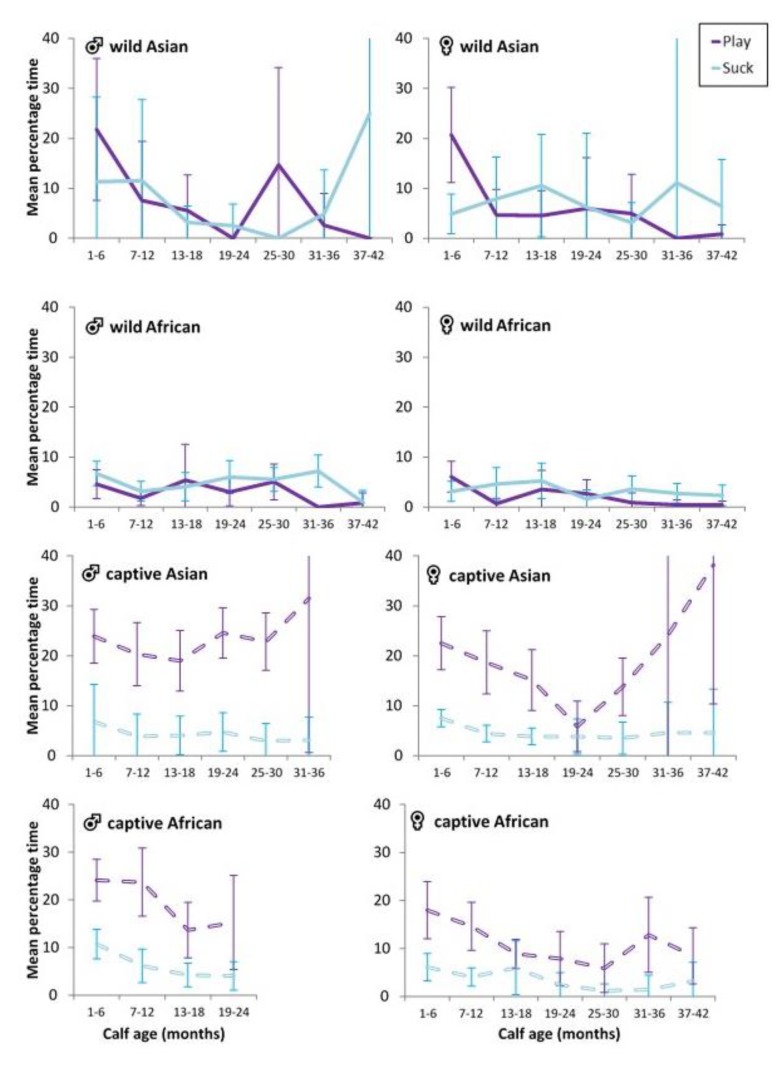
Mean percentage of observation time (±95%CI) in play and suckling interactions by context and sex for calves from birth to 3.5 years.

We anticipated that play would be associated, at least in part, with the energy derived from milk in early life and would decline with weaning, which tends to occur after 24 months and between 3 and 5 years of age [9,10]. Overall correlations between suckling interactions and total time spent in play were generally absent (Table 6), with the exception of a weak association for captive African male calves and a slightly stronger association for captive African female calves. For wild African calves, no clear ‘weaning trough’ in play or suckling was observed at the median reconception age of 25–36 months [10]. Wild Asian males tended to show a drop at 19–24 months as well as 31–36 months (Figure 2). For female wild Asian calves, there did appear to be a ‘weaning trough’ in play around 31–36 months, somewhat later than the decline observed in wild African females. For each captive calf, the percentage of time in play was unrelated to suckling interactions for that age. 

**Figure 3:**
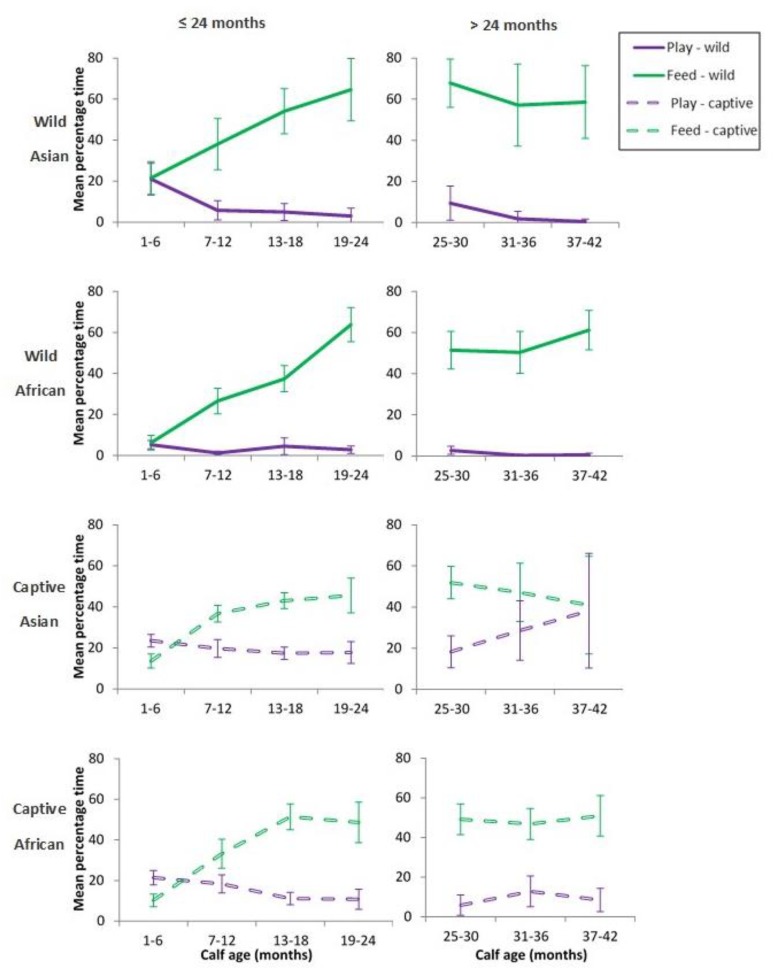
The relationship between the mean percentage of observation time (±95%CI) in total play and independent feeding by context from birth to 3.5 years. Charts separated within context, for ≤24 months and for >24 months.

The lack of any clear association between play and maternally-derived energy suggested that elephant calves were fuelling any extra costs of play via an increase in independent feeding or a decrease in time spent moving. Time spent in independent feeding increased with age (see Figure 3) and was negatively correlated with play in all contexts and for both sexes (Table 6), with the exception of male wild African calves where time spent feeding was only weakly associated with overall play (Table 6). Since the first 24 months were assumed to be the key period of calf dependence on mothers for energy intake [9], we compared time spent in play with that for feeding separately for <24 months and >24 months (Figure 3). Wild Asian and African calves followed similar patterns for play and independent feeding. Time spent in independent feeding increased with calf age and negatively co-varied with play only for the first 24 months of life. For calves older than 24 months, this inverse relationship weakened, and the two behaviours became more concordant. Time spent moving and resting showed no associations, positive or negative with play (Table 6). 

## 4. Discussion 

As a baseline for further explorations of the development and functions of play both in wild and captive elephants and for understanding play’s potential implications for welfare, we show that the general patterns of play by age and sex were similar between species both in the wild and in captivity, despite a small sample of captive calves. We hypothesised that wild calves would play more. The reverse was true however, and captive calves spent more time playing than did wild calves. This pattern was found in both species, and in both social and non-social play. Other studies have also found that play is less frequent in wild populations than in captive groups (e.g., common marmosets [62]). Abundant energy and lack of time constraints due to not needing to forage, move or be vigilant for predators mean that more time and capacity for play are available in captivity. 

Individual calf ID was a significant influence on the time spent in play contributing to these overall patterns, and thus conclusions from the tiny captive sample about wellbeing need to be made with caution. However, despite high individual variation, calf age and context still significantly influenced play behaviours. Captive calves across all ages spent up to an average of 22% of their diurnal time in play, while wild calves played for about 5% (African) to 15% (Asian) of their day. 

The next prediction, that captive calves used behaviour such as non-social play to compensate for a lack of diverse social partners, was also unsupported. While non-social play was again higher in captivity than that in the wild for both species, it was not compensating for any deficit of social play nor did these two types of play co-vary. In wild African calves, almost all play was social, while lone or object play was only rarely observed after the first 12 months. In contrast, wild Asian calf play contained both social and non-social forms. Wild and captive Asian calves engaged in roughly equal proportions of social and non-social play. For all calves, non-social play declined with age with the highest levels of play exploration and lone-locomotor play seen in calves under 6 months of age. The decline in all types of play with increasing age was more apparent in wild Asian than in wild African calves, while play was both less frequent and more constant in early life for African calves. 

The factors that enable or constrain play are various: energetics, partner availability for social play, mood, temperament, distraction by other activities or risks, and in captivity, management and visitor regimes. The high time spent in play among the captive calves, given that they were matched in age with their wild counterparts, requires consideration. Is this a reflection of high levels of welfare and wellbeing, is it a consequence of needing to spend less time on other “maintenance” activities, or did it reflect the constant availability of at least some attractive play partners?

Play partner availability, taken here as calves <60 months of age [10], is a moveable feast for wild elephants with fission-fusion sociality. The family units of the Amboseli elephants were larger (mean FU size = 11 individuals, range = 2–30, mean N calves <60 months per family = 3.59, *N* = 53, AERP long-term data [60]) by comparison to those for wild Asian elephants (mean FU size = 3.07 individuals, range = 2–17, mean N calves <60 months per family = 1.2, *N* = 1366 aggregations [63,64]). Given that African elephant families can associate with other families, changing on a daily or even hourly basis, and that the mean overall group size during the multi-year study period was 26 ± 66 (±SD), wild African calves could have had up to seven different, and dynamically changing, playmates available over a day. Wild Asian calves, sampled during two dry seasons with median group sizes of 6–8 individuals, had 1 to 3 playmates available, again changing over the day [60]. While the captive African calves in this study had as many as six potential playmates available, access was constrained by management practices. Captive Asian calves had at most three other calves <60 months but most still had one or two constantly available potential partners. Thus, while wild African calves could experience a number of different partners, these were available for widely varying amounts of time and possibly not nearby when demands from other activities were low enough to permit play. Captive African calves experienced several partners who were constantly available and unconstrained by competing activity demands. Wild Asian calves had few potential partners who also changed over time, while captive Asian calves again had several, consistent partners. The difference between contexts in partner availability is not necessarily just one of numbers; knowing partners well over long periods might have encouraged more play, while seeking novelty in play partners may be constrained by infrequent opportunities for encountering new playmates. Burghardt [1] suggests that the increased availability of nearby play-partners, due to close proximity in restricted environments with little other stimulation, may facilitate social play in captive animals. The nature of play partners available in captivity is likely to be important for social learning and social stimulus for both male and female calves and needs further exploration. 

While sex differences were not marked in this study due to small sample sizes, they have been reported previously [8,9,10]. For future investigations, we illustrate some sex-specific generalities here (see Figure 1). Male elephants under 6 months of age, and especially those in captivity, spent the most time in social play. Young male elephants use play as an opportunity to experience diverse and novel social partners outwith their own family [8,9]. For males, when immature behaviours are prolonged into later ages, problems can arise from not learning other age-appropriate behaviours [65,66]. Therefore play may be especially important in enabling animals to experience the unexpected in their environments [15,16,67]. Seeking out novel age-mates for social opportunities that are not available via usual companions has been shown to be important in other species such as chimpanzees [68] and dolphins [67,69]. Self-assessment against a variety of “others” as well as gaining “other” knowledge provides for immediate and future encounters, especially potentially dangerous competitive interactions at later ages [1,70]. Male elephants may be using play with strangers as risk-learning in what are low-risk encounters. Such strategies, while limited in current captive contexts, may have been partially compensated for by the high amounts of time devoted to play—play as practice and physical training [1] rather than for managing risk or the unexpected [16]. For females, an increase in mean time spent in social play among captive Asian calves at 3–5 years might be explained as the age when females start to engage in allomothering play with newly available younger calves (see also Lee et al. [10] for wild African calves). 

Only when conditions allow can wild calves engage in certain activities, as they are required to coordinate activities with their mothers and other family members, or risk becoming lost and as a consequence, risk death. Evidence of coordination between activities was however lacking, which was unexpected. Negative associations between time spent in maintenance activities and time spent playing in early life (<24 months) were expected since the prevalence of one behaviour excludes the occurrence of another, but this was only the case for feeding and not for moving or resting. By 24 months of age, independent feeding by wild calves increased to over 50% of the day, leaving less time for play [60,71]. In the first 24 months, time spent in independent feeding negatively co-varied with play but after this age, no relationship was found between play rates and age-specific energy intake from feeding. The younger age groups, and we suggest captive calves, are buffered against the energetic costs of play. Weaning is an energetic and social stressor, and correlated play declines have been found in livestock including cattle [72] particularly during separation (artificial weaning) which may be indicative of either depression/anxiety or lack of energy, or both. However, Donaldson et al. [33] hypothesised that play experience might be used as a coping mechanism, particularly during weaning in domestic piglets. We found little evidence that play declined in the age classes vulnerable to weaning, suggesting that any “weaning troughs” were obscured by variation across individuals both in age at weaning and by the complex nature of that process. 

As would be expected, calves spent less time independently feeding in captivity than did wild calves, probably due to higher quality, easy-accessed foods provided by keepers. Both the time required for foraging and any drive to forage would be reduced from the 16–19 h per day seen in wild contexts [73,74], enhancing the potential for play in captivity since feeding is a constraint that obviously affects the time available for activities such as play in wild calves. In addition to contributing to debates over the preponderance of obese elephants in captivity [75], low time spent foraging opens the question of ‘what to do with your day if you do not need to collect and process food?’ Calves in captivity may be filling this ‘spare time’ with play at least for the first two years of life and in doing so may elude boredom, where boredom represents a lack of arousal, low affect and low motivation for experiences [76,77,78]. A higher amount of spare time may also explain why captive play does not drop off as much after 3–5 years as is seen in the wild. 

Non-social play with objects or locomotor play may also be used by captive animals to increase novelty and variability in constant environments and some species show an increase in play when given larger or more complex environments (e.g., domestic piglets [79]; American mink kits [80]). Play does not, however, associate directly or causally with favourable environmental conditions [49] since it is also a function of motivation and opportunities for engaging with challenging novel contexts, as well as conditions of abundant energy and few competing activities as discussed above. Whether play in captive elephants represents adequate welfare, high affect and arousal, or a mechanism to self-simulate, or both, remains to be further explored by more detailed studies of play contexts, durations and sequences. Thus we cannot yet validate play as a measure of either welfare or wellbeing for captive elephant calves [53,56]. While wild individuals playing only infrequently may be those with compromised growth or energy [8,12], whether individual quality is reflected in play rates in captivity has yet to be determined. It is worth noting that no long-term follow-up of the consequences of play for captive calves was possible due to the death of all but two of the subjects before 7 years old. 

Interpretations of the welfare benefits of play need to be made with care, since no single indicator should be used to assess welfare [44,81]. Our results showing increased play in captivity, suggest that the captive study calves had their proximate needs met [75] and were in a ‘relaxed’ state in the sense of being free from challenges such as hunger, predation or heat stresses [41,78]. They had motivation, opportunity, ample energy, and no distracting activities or threats. They therefore engaged in considerable amounts of physically and emotionally stimulating play behaviour during their active periods. The use of play as an indicator of welfare depends on its occurrence in the absence of compromises, but its presence alone is inadequate to demonstrate high welfare [49] nor is intensity [54] sufficient to represent the wealth of experiences derived from play. It is the absence of play that potentially indicates compromised welfare. We should thus be wary of using play as a welfare indicator without understanding the spectrum of both social and non-social play behaviours, especially given the variation in the range of group sizes and compositions that we find among captive elephants worldwide [48].

## 5. Conclusions

Captive elephant calves, ranging in age from birth to five years old, exhibit similar kinds of play to those seen in wild conspecifics: all calves engaged in lone-locomotor, object, and social play. Both Asian and African species played in comparable ways on this gross scale, and with similar tendencies for males to spend somewhat more time in play, and for play to generally decline in frequency with age. Captivity thus was not an obvious constraint on calves’ ability to engage in play, despite limited space, unvaried and restricted companions, and few role models for how to respond to others during play. These conditions produced highly playful immature elephants, who were able to benefit from the physical and social experiences gained during play, at least in early life. 

Weaning was less of a challenge for the captive calves in relation to time spent in play, as might be expected given abundant, easily accessible energy both from well-nourished mothers and from keepers. Weaning is however a long process for wild elephants and is only completed with the birth of the next sibling. In these captive calves, both very long and very short inter-birth intervals were observed as a function of reproductive management. Thus no associations between age, weaning and play emerged. 

Were the captive calves ‘over-playful’? It is unlikely that ‘too much’ play will have costs in the abundantly resourced and protective environments of captivity. But we suggest that the allocation of so much time to play could indicate a lack of alternative physical and social stimuli, and reflect a need for distracting, stimulating activities. Play therefore may not be a very useful indicator of wellbeing and welfare, unless it is absent. 

## Figures and Tables

**Table 1:** Play types in elephants modified from [10,55]. Note that the analysis here was based on the gross categorisations of play types, rather than on the details of descriptions which are provided as background information.

Play Type		Description of Play	Age-Sex Classes (in African Elephants)
Non-social	Lone (L)	Locomotor: Floppy-running, running while swinging ears and head or head low and swinging in mock-charge. Spinning, rocking, kicking, kneeling down on front legs and/or allowing the trunk to flop their own head from a raised position while opening their mouth (Flop-Trunk-on-Head). Often accompanied by ‘play’ (low, pulsating) trumpets. - This can also include tactile lone play in the form of swimming without contact (e.g., ducking, splashing).	All ages; swimming especially seen in adolescent and adult males
	Object (O)	Playfully exploring objects with trunk, mouth, tusks or feet in a vigorous or gentle manner; throwing objects, rolling objects, general intense manipulation of objects. Repeated acts, associated with playful head and ear movements. - This can also include tactile object play in the form of play with mud or water, dust, vegetation or other tactile stimuli.	All ages
Social	Gentle-contact (G)	Climb upon, wiggling, lean on, rub against, roll onto, shove gently, trunk twining and gentle trunk wrestle. Can also include kneeling down on front legs or Flop-Trunk-on-Head. - This can also include tactile play in the form of swimming with gentle-contact (e.g., trunk-twining in water).	Infant and juveniles, often older juvenile females play with young calves
	Escalated-contact (E)	Mount, reaching over the back of another, tail grasping, chase, push vigorously, vigorous sparring head to head. Trunk in relaxed position. - This can also include tactile play in the form of swimming with escalated-contact (e.g., wrestling in water).	Juvenile and adolescent males and females, adult; swimming especially seen in adolescent and adult males

**Table 2:** Number of individual wild Asian calves sampled, by known sex and age category. Parenthesis = total number of 10 min focal video samples for that age-sex class. Total N calves = 101, median N focals per calf = 2, range = 1–5 across age categories.

Age Category by Sex						
Age Category	1–6 Months	7–12 Months	13–18 Months	19–24 Months	3–5 Years	Total
Male	11 (87)	7 (72)	13 (127)	6 (63)	18 (121)	38 (470)
Female	25 (270)	11 (96)	19 (150)	7 (65)	20 (189)	50 (770)

**Table 3:** Number of wild African calves sampled from 1982 to 1984, by sex and age category. Parenthesis = total number of 60 min continuous focal samples by age–sex class. Total N calves = 129, median N focals per calf = 2, min = 1, max = 5.

Age Category by Sex						
Age Category	1–6 Months	7–12 Months	13–18 Months	19–24 Months	3–5 Years	Overall
Male	24 (355)	24 (311)	25 (274)	12 (125)	52 (551)	58 (1606)
Female	23 (481)	25 (269)	18 (196)	15 (171)	65 (748)	72 (1852)

**Table 4:** Number of 5 min interval scans from all-day focal samples for individual captive calf at each age from birth (from 25 min scans for the older African and Asian juveniles).

**Asian Calf ID and Sex by Age**						
**Age Category**	**1–6 Months**	**7–12 Months**	**13–18 Months**	**19–24 Months**	**3–5 Years**	**Total**
Raman ♂	318	56	0	0	0	374
Nayan ♂	1328	912	543	348	559	3690
Jamilah ♀	1110	511	351	410	284	2666
Hari ♂	750	274	263	0	0	1287
Bala ♀	603	277	262	0	0	1142
Scott ♂	586	264	228	0	0	1078
Gheta ♀	0	0	0	0	414	414
Ned ♂	0	0	61	164	171	396
**African Calf ID and Sex by Age**						
**Age Category**	**1–6 Months**	**7–12 Months**	**13–18 Months**	**19–24 Months**	**3–5 Years**	**Total**
Mchumba ♂	684	410	263	224	0	1581
Mansi ♀	0	565	430	332	147	1474
Jaluka ♀	686	185	281	181	0	1333
Tammi’s ♀	128	0	0	0	0	128
Impi ♂	412	191	224	67	0	894
Etana ♀	0	0	0	0	756	756
Uzuri ♀	0	0	0	0	525	525

**Table 5:** Mean, Median, Interquartile Range (IQR) and 95% Confidence Interval (CI) for percentages of total activity budget spent in social, non-social and total play for calves <24 months, by context. Total N male, N female sampled in brackets.

Percent Play	Social Play				Non-Social Play				Total play			
	Mean %	Median %	IQR %	95% CI	Mean %	Median %	IQR %	95% CI	Mean %	Median %	IQR %	95% CI
**Wild Asian (N = 39, 56)**	4.89	0.00	0.00	2.69–7.09	6.19	0.00	0.00	3.72–8.66	11.08	0.00	16.67	7.52–14.63
**Wild African (N = 52, 69)**	2.82	0.00	7.14	1.83–3.85	0.70	0.00	0.00	0.21–1.20	3.54	0.00	7.69	2.36–4.73
**Captive Asian (N = 5, 3)**	9.17	8.66	13.16	7.97–10.39	11.29	9.76	11.85	9.87–12.70	20.46	18.75	14.77	18.55–22.37
**Captive African (N =2, 5)**	5.71	3.92	7.84	4.56–6.85	10.34	10.14	9.49	8.81–11.85	16.04	14.71	15.79	13.94–18.14

**Table 6:** Spearman correlations (*r*_s_) between major activities and total play across age categories of calves, separated by context and sex. Only correlations with probability greater than 0.005 are highlighted due to repeated tests between the four behaviours.

Context	Behaviour	Correlation Coefficient r_s_				
		Rest	Move	Suck	Play	N
**Asian wild male**	Feed	**−0.328 (*p* = 0.005)**	**−0.471 (*p* < 0.001)**	−0.206 (*p* = 0.081)	**−0.417 (*p <* 0.001)**	73
	Rest		0.150 (*p* = 0.204)	−0.167 (*p* = 0.158)	−0.003 (*p* = 0.983)	73
	Move			−0.052 (*p* = 0.664)	−0.112 (*p* = 0.347)	73
	Suck				0.047 (*p* = 0.695)	73
**African wild male**	Feed	**−0.808 (*p* < 0.001)**	**−0.376 (*p* < 0.001)**	−0.016 (*p* = 0.253)	−0.159 (*p* = 0.085)	118
	Rest		−0.021 (*p* = 0.820)	0.028 (*p* = 0.764)	0.058 (*p* = 0.529)	118
	Move			−0.120 (*p* = 0.196)	−0.091 (*p* = 0.325)	118
	Suck				−0.033 (*p* = 0.725)	118
**Asian captive male**	Feed	**−0.541 (*p* < 0.001)**	**−0.403 (*p* < 0.001)**	**−0.286 (*p* = 0.003)**	**−0.387 (*p* < 0.001)**	106
	Rest		−0.209 (*p* = 0.032)	0.237 (*p* = 0.014)	−0.078 (*p* = 0.428)	106
	Move			−0.037 (*p* = 0.708)	−0.107 (*p* = 0.277)	106
	Suck				−0.056 (*p* = 0.568)	106
**African captive male**	Feed	**−0.665 (*p* < 0.001)**	**−0.511 (*p =* 0.001)**	**−0.758 (*p* < 0.001)**	**−0.460 (*p* = 0.002)**	42
	Rest		0.129 (*p* = 0.416)	**0.426 (*p* = 0.002)**	0.032 (*p* = 0.840)	42
	Move			0.225 (*p* = 0.151)	0.228 (*p* = 0.146)	42
	Suck				0.387 (*p* = 0.011)	2
**Asian wild female**	Feed	**−0.278 (*p* = 0.003)**	**−0.424 (*p* < 0.001)**	−0.052 (*p =* 0.583)	**−0.392 (*p <* 0.001)**	110
	Rest		−0.069 (*p =* 0.472)	0.056 (*p =* 0.556)	0.167 (*p =* 0.078)	110
	Move			−0.137 (*p =* 0.151)	−0.081 (*p =* 0.399)	110
	Suck				0.024 (*p =* 0.801)	110
**African wild female**	Feed	**−0.787 (*p <* 0.001)**	−0.204 (*p =* 0.019)	−0.115 (*p =* 0.187)	**−0.249 (*p =* 0.004)**	130
	Rest		−0.192 (*p =* 0.027)	0.006 (*p =* 0.944)	0.139 (*p =* 0.114)	130
	Move			0.010 (*p =* 0.910)	−0.133 (*p =* 0.130)	130
	Suck				−0.086 (*p =* 0.327)	130
**Asian captive female**	Feed	**−0.570 (*p <* 0.001)**	−0.295 (*p =* 0.016)	**−0.434 (*p <* 0.001)**	**−0.552 (*p <* 0.001)**	66
	Rest		0.068 (*p =* 0.589)	**0.318 (*p =* 0.009)**	−0.105 (*p =* 0.404)	66
	Move			−0.007 (*p =* 0.956)	−0.114 (*p =* 0.362)	66
	Suck				0.023 (*p =* 0.852)	66
**African captive female**	Feed	−0.095 (*p =* 0.423)	**−0.485 (*p <* 0.001)**	**−0.297 (*p =* 0.010)**	**−0.354 (*p =* 0.002)**	74
	Rest		−0.141 (*p =* 0.231)	−0.083 (*p =* 0.481)	−0.130 (*p =* 0.268)	74
	Move			0.170 (*p =* 0.149)	0.020 (*p =* 0.865)	74
	Suck				**0.399 (*p <* 0.001)**	74

## Supplementary Materials

The following are available online at https://www.mdpi.com/2076-2615/10/2/305/s1, Table S1: Full GLMM model with all significant factors and interactions as well as pairwise comparisons between sex, context and age categories shown for total play, Table S2: Full GLMM model for social play, Table S3: Full GLMM model for non-social play, Figure S1: Individual time spent in play for captive Asian elephant calves, Figure S2: Individual time spent in play for captive African elephant calves. Original activity data (excel format) available in https:/datastorre.stir.ac.uk.
