# Supplementary material for: Play in Elephants: Wellbeing, Welfare or Distraction?
Source: Animals (Basel). 2020 Feb 14;10(2):305. doi: 10.3390/ani10020305 (PMC7071055; doi:10.3390/ani10020305)
Supplement: Supplementary file 1 [file animals-10-00305-s001.pdf]

animals

MDPI

Article

Supplementary Materials: Play in Elephants: Wellbeing, Welfare or Distraction?

C. Elizabeth Webber and Phyllis C. Lee *

Table S1. Final General Linear Mixed Model for total-play. Var (ID) = 24.98, $p = 0.003$. Dependent variable was total Play; independent variables were age (categorical, reference category: 1–6 mo), context (reference category: wild Asian), and the interactions between them. Main effects for independent factors presented as F with degrees of freedom for significant factors only, interaction effects between factors (bold) and pairwise comparisons (not bold) are shown as Coefficient (ß) with 95 % confidence Interval. Where overall main effects or interactions were not significant (NS), but interactions were significant within categories or contexts, these were retained. All multiple comparisons and post-hoc pairwise tests used least significant difference to adjust for repeated tests; significance (*p*) associated with pairwise comparisons represents those adjusted for N of comparisons.

Total Play	Main Effect *F* and *p* Value	Coefficient β (95% CI)	*P* Value
Overall model fit	$F_{19, 385} = 10.135, p < 0.001$		
Age	$F_{4, 492} = 11.614, p < 0.001$		
1–6mo–7–12mo		6.532 (3.735 to 9.330)	<0.001
1–6mo–13–18mo		7.878 (5.140 to 10.616)	<0.001
1–6mo–19–24mo		7.957 (4.103 to 10.338)	<0.001
1–6mo–3.5yr		7.221 (4.103 to 10.338)	<0.001
Context	$F_{3, 58} = 26.0337, p < 0.001$		
**Asian captive**		24.770 (18.192 to 31.348)	<0.001
Asian wild–African wild		4.659 (1.869 to 7.448)	0.001
Asian wild–Asian captive		−14.020 (−18.622 to −9.419)	<0.001
Asian wild–African captive		−5.695 (−10.721 to −0.668)	0.027
African wild–Asian captive		−18.679 (−23.195 to −14.162)	<0.001
African wild–African captive		−10.353 (−15.302 to −5.405)	<0.001
Asian captive–African captive		8.326 (2.150 to 14.501)	0.010
Age × context	$F_{12, 540} = 3.696, p < 0.001$		
**1–6mo Asian wild vs all others**		12.308 (6.039 to 18.577)	<0.001
**13–18mo Asian captive vs all others**		−11.243 (−19.563 to −2.923)	0.008
1–6mo–7–12mo × Asian wild		16.494 (10.432 to 22.556)	<0.001
1–6mo–13–18mo × Asian wild		16.233 (11.254 to 21.213)	<0.001

1–6mo–19–24mo × Asian wild	17.440 (11.013 to 23.867)	<0.001
1–6mo–3–5yrs × Asian wild	16.309 (11.747 to 20.871)	<0.001
1–6mo–13–18mo × Asian captive	5.299 (0.209 to 10.389)	0.041
13–18mo–3–5yrs × Asian captive	−7.955 (−14.766 to −1.144)	0.022
1–6mo–13–18mo × African captive	9.267 (2.806 to 15.727)	0.005
1–6mo–19–24mo × African captive	9.318 (2.492 to 16.144)	0.008
1–6mo–3–5yrs × African captive	11.229 (2.501 to 19.956)	0.012
7–12mo–13–18mo × African captive	7.588 (0.950 to 14.226)	0.025
7–12mo–19–24mo × African captive	7.639 (0.619 to 14.660)	0.033
7–12mo–3–5yrs × African captive	9.550 (0.824 to 18.276)	0.032

Table S2. Final General Linear Mixed Model for Social Play. Var (ID) = 2.86, $p = 0.132$. Dependent variable was social play; significant independent variables were age (categorical, reference category: 1–6 mo), context (reference category: wild Asian), sex and the interactions between them. Main effects for independent factors presented as F with degrees of freedom for significant factors only, interaction effects between factors (bold) and pairwise comparisons (not bold) are shown as Coefficient (*ß*) with 95 % confidence Interval. Where overall main effects or interactions were not significant (NS), but interactions were significant within categories or contexts, these were retained. All multiple comparisons and post-hoc pairwise tests used least significant difference to adjust for repeated tests; significance (*p*) associated with pairwise comparisons represents those adjusted for N of comparisons.

Social Play	Main Effect *F* and *p* Value	Coefficient *β* (95% CI)	*p* Value
Overall model fit	$F_{38, 194} = 3.554$, $p < 0.001$		
Context	$F_{3, 37} = 20.282$ $p < 0.001$		
**Asian captive vs all others**		11.304 (5.080 to 16.988)	<0.001
Asian wild–Asian captive		−7.459 (−9.776 to −5.141)	<0.001
Asian wild–African captive		−2.722 (−5.267 to −0.177)	0.037
African wild–Asian captive		−8.002 (−10.209 to −5.796)	<0.001
African wild–African captive		−3.266 (−5.711 to −0.821)	0.010
Asian captive–African captive		4.737 (1.819 to 7.655)	0.003
Age × sex	$F_{4, 513} = 1.015$ $p = 0.399$ (NS)		
**1–6mo × male**		15.432 (2.340 to 28.523)	0.021
**7–12mo × male**		17.668 (4.251 to 31.085)	0.010
**13–18mo × male**		16.534 (3.106 to 29.962)	0.016
**19–24mo × male**		15.363	0.001

		(6.125 to 24.601)	
Age × context	$F_{12, 384}$=2.191 p=0.012		
**1–6mo × Asian wild**		8.128 (1.134 to 15.123)	0.023
Sex × context	$F_{3, 39}$ = 1.515 p = 0.226 (NS)		
**Male × Asian wild**		14.583 (1.715 to 27.450)	0.026
Sex × age (within context)	$F_{11, 607}$ = 1.360 p = 0.187 (NS)		
**Male × 1–6mo (within Asian wild)**		−22.313 (−36.892 to −7.733)	0.003
**Male × 7–12mo (within Asian wild)**		−24.955 (−40.546 to −9.364)	0.002
**Male × 13–18mo (within Asian wild)**		−19.655 (−34.484 to −4.824)	0.009
**Male × 19–24mo (within Asian wild)**		−18.429 (−31.036 to −5.821)	0.004
**Male × 1–6mo (within African wild)**		−15.970 (−30.125 to −1.816)	0.027
**Male × 7–12mo (within African wild)**		−17.117 (−31.694 to −2.541)	0.021
**Male × 13–18mo (within African wild)**		−14.826 (−29.598 to −0.054)	0.049
**Male × 19–24mo (within African wild)**		−15.882 (−27.459 to −4.304)	0.007
**Male × 1–6mo (within Asian captive)**		−12.666 (−25.046 to −0.287)	0.045

Table S3. Final General Linear Mixed Model for Non-social Play. Var (ID) = 12.35, $p > 0.001$. Dependent variable was non-social play; independent variables were age (categorical: 1–6mo as reference category), context (wild Asian as reference category), and the interactions between them. Main effects for independent factors presented as F with degrees of freedom for significant factors only, interaction effects between factors (bold) and pairwise comparisons (not bold) are shown as Coefficient (*ß*) with 95 % confidence Interval. Where overall main effects or interactions were not significant (NS), but interactions were significant within categories or contexts, these were retained. All multiple comparisons and post-hoc pairwise tests used least significant difference to adjust for repeated tests; significance (*p*) associated with pairwise comparisons represents those adjusted for N of comparisons.

**Non-Social Play**	**Main Effect *F* and *p* value**	**Coefficient *β* (95% CI)**	***p* value**
Overall model fit	$F_{19, 445}$ = 9.918, $p < 0.001$		
Age	$F_{4, 528}$ = 18.537, $p < 0.001$		
**1–6mo–7–12mo**		6.007 (4.166 to 7.847)	<0.001
**1–6mo–13–18mo**		6.697 (4.896 to 8.498)	<0.001
**1–6mo–19–24mo**		5.872 (3.786 to 7.958)	<0.001
**1–6mo–3–5yr**		5.752 (3.672 to 7.831)	<0.001
Context	$F_{3, 66}$ = 18.146, $p < 0.001$		
African wild		13.852 (9.420 to 18.284)	<0.001
**Asian wild–frican wild**		3.504 (1.639 to 5.369)	<0.001
**Asian wild–Asian captive**		−6.356 (−9.508 to −3.205)	<0.001
**Asian wild–African captive**		−3.897	0.027

		(−7.334 to −0.461)	
**African wild–Asian captive**		−9.860<br>(−12.957 to −6.764)	<0.001
**African wild–African captive**		−7.401<br>(−10.787 to −4.015)	<0.001
Age × context	$F_{12, 555} = 3.302$, $p < 0.001$		
1–6mo Asian wild		8.626<br>(4.466 to 12.789)	<0.001
1–6mo African captive		9.311<br>(2.799 to 15.824)	0.005
7–12mo Asian captive		−6.723<br>(−12.080 to −1.366)	0.014
13–18mo Asian captive		−7.735<br>(−13.233 to −2.236)	0.006
**1–6mo–7–12mo Asian wild**		10.448<br>(6.440 to 14.456)	<0.001
**1–6mo–13–18mo Asian wild**		9.007<br>(5.719 to 12.296)	<0.001
**1–6mo–19–24mo Asian wild**		8.501<br>(4.251 to 12.752)	<0.001
**1–6mo–3–5yrs Asian wild**		10.172<br>(7.147 to 13.198)	<0.001
**1–6mo–7–12mo Asian captive**		7.269<br>(4.166 to 10.371)	<0.001
**1–6mo–13–18mo Asian captive**		7.519<br>(4.182 to 10.856)	<0.001
**1–6mo–19–24mo Asian captive**		4.975<br>(0.972 to 8.979)	0.015
**7–12mo–3–5yrs Asian captive**		−6.839<br>(−11.287 to −2.391)	0.003
**13–18mo–3–5yrs Asian captive**		−7.090<br>(−11.590 to −2.590)	0.002
**19–24mo–3–5yrs Asian captive**		−4.546<br>(−9.080 to −0.012)	0.049
**1–6mo–7–12mo African captive**		4.648<br>(0.393 to 8.903)	0.032
**1–6mo–13–18mo African captive**		9.359<br>(5.119 to 13.598)	<0.001
**1–6mo–19–24mo African captive**		8.303<br>(3.824 to 12.782)	<0.001
**1–6mo–3–5yrs African captive**		10.858<br>(5.003 to 16.713)	<0.001
**7–12mo–13–18mo African captive**		4.711<br>(0.362 to 9.060)	0.034
**7–12mo–3–5yrs African captive**		6.210<br>(0.373 to 12.047)	0.037

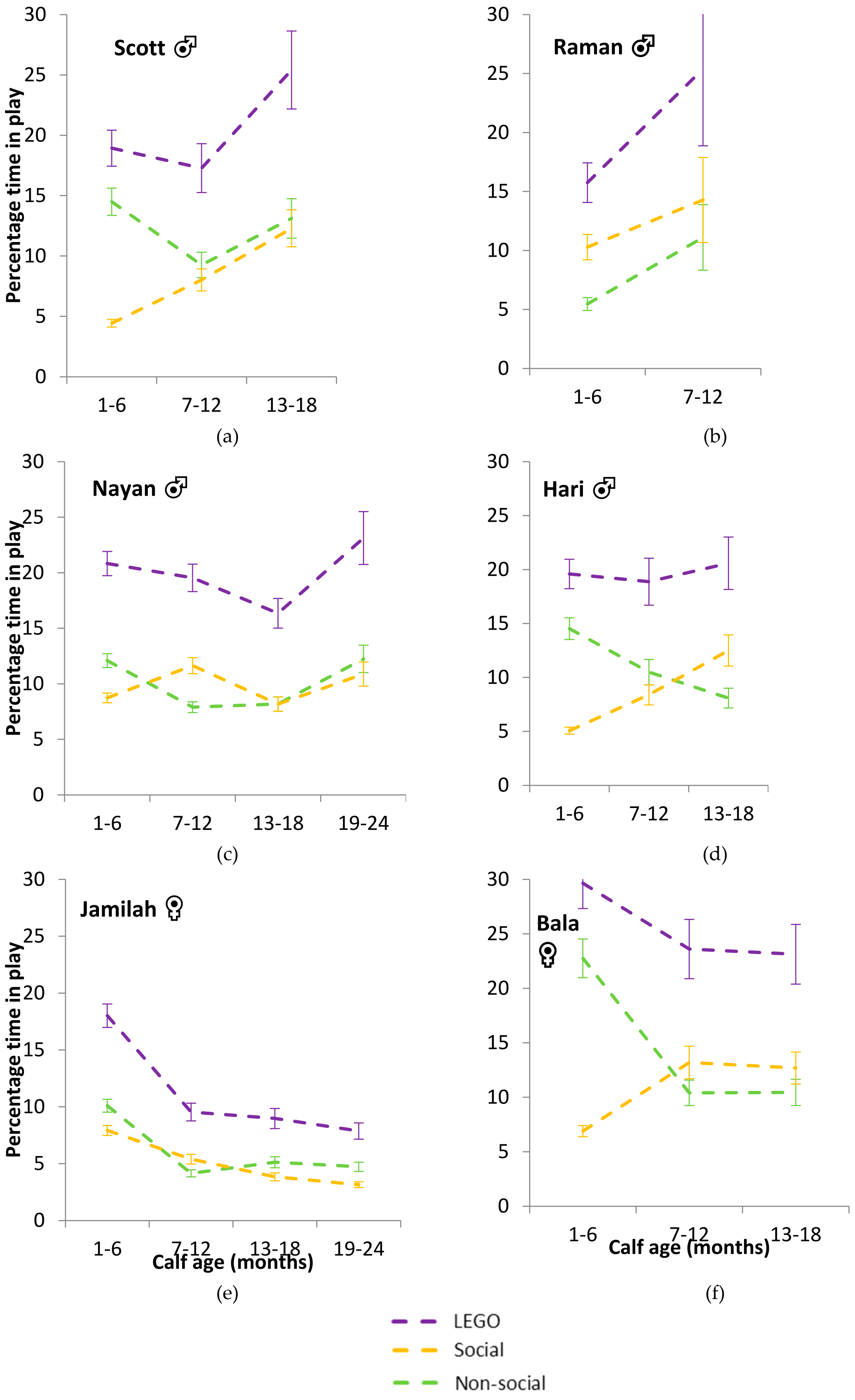

Figure S1. Play categories for individual captive Asian calves followed from birth to 18+ months of age (or death); (a) Scott, (b) Raman, (c) Nayan, (d) Hari, (e) Jamilah, (f) Bala. Social, non-social and LEGO (TOTAL) play types. Data from scans at 5min intervals from 10min focal observations. Binomial 95%CI.

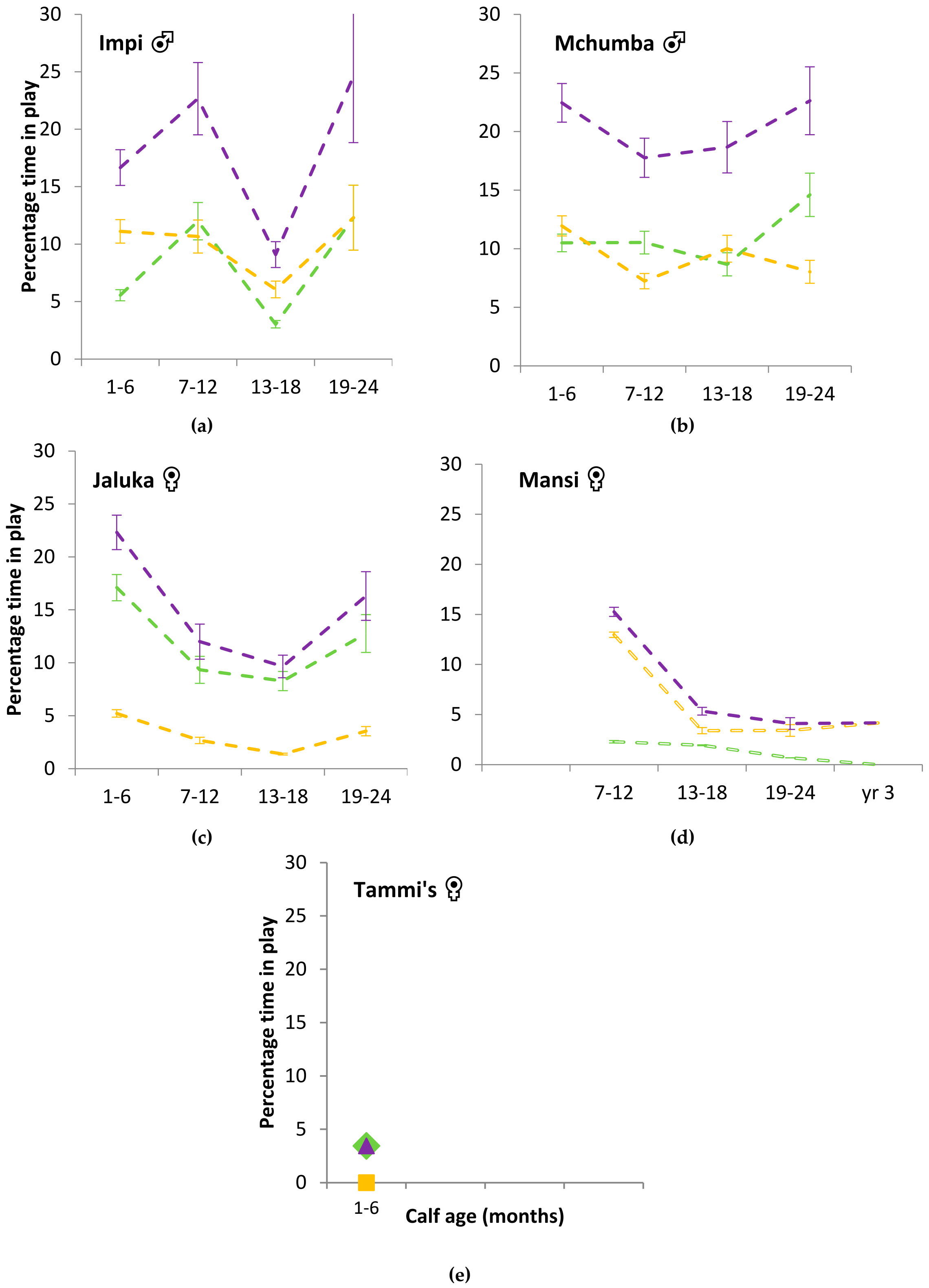

Figure S2. Play categories for individual captive African calves followed from birth to 18+ months (or death); (a) Impi, (b) Mchumba, (c) Jaluka, (d) Mansi, (e) Tammi's. Social, non-social and Total play types (legend as Supp Fig 1). Data from scans at 5min intervals from 10min focal observations. For Tammi's calf, the two incidences of play were trunk-behaviours. Binomial 95% CI.

© 2020 by the authors. Licensee MDPI, Basel, Switzerland. This article is an open access article distributed under the terms and conditions of the Creative Commons Attribution (CC BY) license (http://creativecommons.org/licenses/by/4.0/).
